# Citizens as Active Participants in Integrated Care: Challenging the Field’s Dominant Paradigms

**DOI:** 10.5334/ijic.4202

**Published:** 2019-03-14

**Authors:** Ludo Glimmerveen, Henk Nies, Sierk Ybema

**Affiliations:** 1 Vrije Universiteit Amsterdam, NL; 2 Vilans, Centre of Expertise for Long-term Care, NL

**Keywords:** integrated care, co-production, citizen participation, public engagement, informal care, user involvement

## Abstract

Policy makers, practitioners and academics often claim that care users and other citizens should be ‘at the center’ of care integration pursuits. Nonetheless, the field of integrated care tends to approach these constituents as passive recipients of professional and managerial efforts. This paper critically reflects on this discrepancy, which, we contend, indicates both a key objective and an ongoing challenge of care integration; i.e., the need to reconcile (1) the professional, organizational and institutional frameworks by which care work is structured with (2) the diversity and diffuseness that is inherent to pursuits of active user and citizen participation. By identifying four organizational tensions that result from this challenge, we raise questions about whose knowledge counts (lay/professional), who is in control (local/central), who participates (inclusion/exclusion) and whose interests matter (civic/organizational). By making explicit what so often remains obscured in the literature, we enable actors to more effectively address these tensions in their pursuits of care integration. In turn, we are able to generate a more realistic outlook on the opportunities, limitations and pitfalls of citizen participation.

## Introduction

The field of integrated care has an ambiguous relationship with citizens and communities, i.e., with those whose lives may be affected by how services are organized. Both integrated care policy and literature consistently stress the importance of ‘putting the individual at the centre of all interventions’ [1]. Recognizing that care services ought to reflect ‘the needs of the local population’ [2], scholars and policy makers often call for ‘stronger citizen’s participation’ [3] in their governance and delivery. Such ambitions have resulted in a vast range of efforts that are often referred to as co-production—an umbrella term for situations in which care users and other citizens ‘contribute to the provision of health [or social care] services as partners of professional providers’ [4]. For example, co-production is often pursued in partnerships between individual users and professionals [5], but also among clients’ family members in nursing homes [6] or in local rural communities [7,8]. While co-production can encompass collaboration within the actual delivery of care, it can also entail more ‘upstream’ participation of users and citizens; i.e., as they co-design services together with professionals, managers or policy makers, and take part in ‘identify[ing] the kinds of problems to which a service responds, rather than just giving people a say in the answers to pre-defined problems’ [9]. Despite this emphasis on their participation, however, these same ‘individuals’, ‘local populations’ and ‘citizens’ tend to remain remarkably marginal within the dominant frameworks and models that shape our thinking about integrated care, whether implicitly or explicitly positioned as passive recipients of professional or managerial efforts at integration. By continuing to be dominated by established institutional, organizational and professional paradigms—each with their own assumptions regarding who is ‘in charge’ of integrating services—integrated care has arguably become ‘too much [of] a professionals’ concept’ [10], being pursued *for* citizens while insufficiently acknowledging the potential contribution made *by* citizens.

In this paper, we zoom in on how pursuits of citizen participation challenge established conceptualizations of integrated care. We particularly focus on the organizational tensions and ambiguities that we consider intrinsic to all pursuits of participation. This allows us to move beyond simplified claims that either place users and citizens ‘in the driver’s seat’ or that, alternatively, treat them as mere targets of integration. By reflecting on these organizational tensions, we expose the power dynamics that often remain implicit within integrated care literature: to what extent are users and other citizens able to actually influence the outcomes of service integration? Our argument is inspired by our own research at the intersection of participation and care integration [11]. In our studies, we consistently witnessed policy makers, managers, professionals and citizens navigating multiple organizational tensions as they faced disparate positions, perspectives and interests within and between groups of actors. In this article, we connect these observations to a broader collection of studies on user and citizen participation. Building on this literature, we demonstrate how participation may catalyze as well as complicate pursuits of integration, as it challenges established divisions of roles and responsibilities on both individual and collective levels of service governance and delivery.

While it is surely not our aim to comprehensively cover extant literature across these different levels of integration, we do want to highlight a shared challenge that underlies such diverse efforts; namely, the need to reconcile (a) the diversity of citizens’ concerns and their voluntary involvement—which is often ‘not institutionally, vocationally nor financially bound’ and therefore ‘difficult to control and to steer’ [12]—with (b) the professional, organizational and institutional frameworks on which practitioners, managers and policy makers draw when structuring their work. Put differently: how do we accommodate the diversity and diffuseness of citizens’ ‘life-worlds’ while still relying on the systems that support us in the organization of high-quality, equitable and cost-efficient services? As we will elaborate on in this paper, this crucial challenge too often remains implicit within discussions of integrated care, obscuring a number of key tensions that need to be dealt with when citizens become active participants in care integration. In contrast, our analysis places these tensions front and center by conceptually and practically exploring how they challenge our way of thinking about citizen positions in co-designing and co-producing integrated care.

Although triggered by our own empirical encounters, our argument rests on a conceptual review [13] in which we connect the literature on care integration and citizen participation. Instead of doing a systematic review—which others have done before us (see [14,15,16] on integrated care; or [17,18] on citizen participation)—a conceptual review allows us to more flexibly capture the intricate dilemmas at the intersection of participation and integration. As a starting point for our review, we made a selection of the International Journal of Integrated Care’s most-cited articles (e.g., [1,2,3,19]), indicating the degree to which these have impacted contemporary thinking on integrated care. Focusing on these publications, we first analyze scholars’ explicit and implicit positioning of citizens. Then, we supplement with and compare this literature to studies that explicitly focus on user and citizen participation at various levels of service organization. By combining these literatures, we are then able to identify four key organizational tensions that become increasingly salient when citizens adopt a more prominent role in care integration pursuits; i.e., those pertaining to dilemmas involving (1) whose knowledge counts (lay/professional), (2) how power is distributed (local/central), (3) who participates (inclusion/exclusion) and (4) whose interests prevail (organizational/civic). While often remaining implicit in extant literature and in instances in which care is integrated *for* citizens, we contend that making these tensions explicit provides a more realistic outlook on the opportunities, limitations and pitfalls of citizen participation in care integration. By zooming in on how these tensions are addressed, we are able to highlight people’s underlying assumptions regarding who is in charge of and responsible for care integration. Accordingly, we contend that these assumptions should be made more explicit within current conceptualizations of integrated care. As a result, this paper revolves around the following question: how can we incorporate the organizational dynamics of user and citizen participation when thinking about integrated care? In essence, we explore what it means to not only integrate organizational or professional systems, but also to align these with individuals’ diverse and diffuse life-worlds.

Below, we continue by discussing the ambiguous positioning of users and citizens within integrated care research and theorizing. Then, we zoom in on the organizational tensions that help us to better understand and, subsequently, deal with this ambiguity.

## Integrated care and the ambiguous position of users and other citizens

Progressively moving away from the idea that lay involvement is restricted to care users ‘patiently’ awaiting professional treatment [20,21], scholars within [10,22,23] and beyond [24,25,26] the field of integrated care have highlighted the increasing centrality of citizens and communities in organizing care services. In itself, the emergence of integrated care as a policy imperative signifies a growing concern with ‘the whole person’ within the organization of care services. Following from the realization that people’s health and wellbeing are, for a large part, shaped by their broader social environment [10,20], improved connectivity between medical and social domains has become a defining objective within many pursuits of integrated care [2,27,28]. The underlying implication is that care services need to be attuned to the ‘life-world’ of service users and citizens—i.e., to their particular experience of their situation—and that professional and organizational efforts need to be aligned with people’s private responsibilities.

Whereas the inclusion of (professional) social care services has become part and parcel of many integrated care approaches (see e.g., [29,30]), scholars and policy makers have argued for further expansion of the scope of integration by ‘actively involv[ing] and empower[ing] the people it is serving – both on an individual and collective level’ [20]. Indeed, co-producing public services with citizens has become an increasingly prominent policy imperative internationally (e.g., in England [31], the Netherlands [32,33], Denmark [34] and the United States [35]). Instead of only being *passive objects* of professional or managerial efforts, citizens are also increasingly becoming *active subjects* on various levels of care integration [10]. Often, such policy ambitions imply that users and other citizens ought to have a more pronounced say in how services are designed and delivered, i.e., granting them more influence over decision making.

At the same time, however, a substantial amount of research highlights a discrepancy between such policy ambitions and citizens’ actual participation in organizing services (e.g., [19,22,36]). At the level of individual care trajectories, for example, scholars have criticized care professionals for failing to appreciate the user perspective [19] and for treating a care user’s family as an opponent or a nuisance instead of as a partner [1]. Moreover, objectives of user empowerment—e.g., enabling participants to ‘shape their own lives rather than hav[ing] them shaped by others’ [36]—often continue to be determined by medical-professional regimes that emphasize disease control [36]. At policy and governance levels, scholars have seen managers and policy makers engage in ‘tokenistic’ or even ‘manipulative’ efforts at citizen participation [37,38,39]. Even when participatory efforts are supported by a broad range of stakeholders, they often still fail to deliver on the sought-after results [40,41]. Apparently, the often-cited imperative to ‘place individuals at the center’ does not necessarily lead to a redistribution of power and/or responsibilities within actual processes of care integration [11].

A key question that has not received due attention, then, is why active user and citizen participation has proven so hard to realize. We contend that the limited success of participatory ambitions can, at least in part, be traced back to the field of integrated care itself, in particular to how the field conceptualizes care integration. Despite recurrent calls to foster citizen participation, the models and frameworks that guide pursuits of integration are still dominated by institutional and professional paradigms. Accordingly, it often remains unclear whether participation is an add-on to an otherwise systems-driven attempt at integration or, alternatively, a more fundamental alteration of citizens’ position in the governance and delivery of care services. For example, while Kodner and Spreeuwenberg are critical of ‘systems- or organisation-driven’ efforts at care integration in their seminal article [19], their own conceptualization of integrated care leaves limited space for citizens’ active participation. By defining it as a ‘set of methods and models on the funding, administrative, organisational, service delivery and clinical levels designed to create connectivity, alignment and collaboration within and between the cure and care sectors’ [19], integrated care continues to be an issue of policy makers, managers and professionals. Similarly, Valentijn et al.’s often-cited conceptual framework highlights the centrality of people’s ‘personal preferences, needs, and values’ [2] as well as ‘the needs of the local population’ [2]. They implicitly position citizens as passive recipients care is being integrated *for*. Stating that professionals ‘have a collective responsibility to provide a continuous, comprehensive, and coordinated continuum of care to a population’ [2], which requires the ‘collective action of organisations across the entire care continuum’ [2], they also portray care integration as a predominantly professional endeavor. Moreover, the various labels they assign to the different levels of integration—distinguishing between clinical, professional, organizational and system-level integration—are also indicative of the assumed professional and institutional character. While we do not question the usefulness and authenticity of these various authors’ claims regarding the centrality of ‘patients’ and ‘populations’ to integrated care, their frameworks simultaneously insinuate that care users are passive beneficiaries of professional services [42,43].

To a fair extent, we believe that the ambiguous position of citizens is inevitable in pursuits of integrated care [11]. Although often promising to put users and citizens in the ‘driver’s seat’, actors involved in care integration face a broad set of other, often competing systemic demands and policy imperatives. Care integration comes with the challenge of reconciling, on the one hand, people’s life-worlds—the need to accommodate the diversity of what matters to individual people [36] and the diffuse and potentially unstable dynamics of their situation [44]—and, on the other hand, the technical rationalities of the systems (either professional, organizational or otherwise) through which care services are organized. That such systems have become too detached from people’s life-worlds, ergo, obstructing the pursuit of ‘what really matters’ [45] in care delivery, while also inhibiting users’ and citizens’ opportunities to take responsibility of how care is being organized are common critiques. Even given such a reality, however, such systems, structures, models and guidelines remain essential to organizing equitable, accountable and high-quality services [33]. Claims of putting citizens in the ‘driver’s seat’, consequently, only create a caricature of a key challenge within integrated care: the ongoing need to align these organizational systems with the particularities of people’s life-worlds.

We propose a conceptual recalibration of citizens’ position in care integration—one that acknowledges the critical, ongoing challenge of aligning systems and life-worlds [46] throughout the processes of integration. By making this challenge more explicit, we improve our ability to reflect on a key conundrum surrounding citizen participation in integrated care: to what extent do professionals, managers and policy makers ‘structure’ participation or, alternatively, are users and other citizens granted the ability to shape the character of the services they (potentially) use? While we may intuitively see the latter as a desirable guiding principle, the reality is often not as straightforward in practice. Person-centered organizing, particularly when users and other citizens become active participants in such efforts, presents us with potential trade-offs and a need to balance or align competing considerations. While it is tempting to promote ambitions that place citizens in ‘the driver’s seat’, the question of who is in charge of care integration remains more complex. In what follows, we explore in more detail what this implies for the position of citizens in integrated care. Acknowledging that participation pursuits often challenge established distributions of control and responsibilities, we highlight the delicate power relations between the various actors involved—an issue that is too often neglected or implicit within integrated care research.

## Organizing citizen participation in care integration: navigating four tensions

If a key challenge in the pursuit of integrated care is, indeed, the ongoing need to align systems and life-worlds, then what does this mean for the organizational dynamics of such pursuits? To answer this question, we will now discuss four key organizational tensions that become increasingly salient within participatory care-integration efforts (see Table 1). By moving back and forth between our own research experiences and extant literature on both participation and care integration, we found these four tensions to underlie many of the challenges of realizing effective participation. While we do not claim this list to be exhaustive, we do believe that these four tensions are broadly applicable. Even if different dynamics and challenges exist across different levels of integration (see, e.g., [47] for a review of the differences between patient and public involvement), each of these tensions may surface at any level of organizing and within vastly different participatory initiatives. By focusing on these tensions, we contribute to an understanding of why efforts at integration may continue to fall short of their ambitions to develop more person-centered and population-based services. By explicating what so often remains implicit in extant literature, we enable actors to more effectively identify and address these organizational tensions in their pursuits of user and citizen participation—including the power dynamics that often shape their manifestation.

**Table 1:** Overview of tensions.

	Domain	Source of tension

1.	Expertise	The need to reconcile lay and professional knowledge
2.	Control	The need to reconcile local alignment and central coordination
3.	Inclusion and exclusion	The need to reconcile citizens’ diversity and their formation as participants
4.	Interests	The need to reconcile the concerns of citizens and organizational members

In what follows, we discuss each tension by first illustrating how it surfaced within our own ethnographic studies on (tension 1) user participation in a nursing home and (tensions 2, 3 and 4) the community-participation platforms that surrounded a rural care facility. Throughout these studies, conducted between 2013 and 2015, we investigated the contentious processes through which professional provider organizations translated their own policy imperative—to engage with citizens—into concrete participatory practices (see [11] for more background information). The four illustrations we have drawn from this research involve a variety of issues, ranging from more-fundamental aspects of local service design (e.g., deciding whether a care facility stays open as a ‘joint venture’ between a professional provider organization and a local community) to somewhat-smaller issues pertaining to everyday service provision (e.g., how to improve the meals provided in a nursing home). Nonetheless, each example revolves around an attempt to integrate ‘lay’ perspectives and concerns within the professional organization of services. Moreover, each highlights the intricate challenges of such pursuits, even when they pertain to issues that may seem fairly straightforward or trivial at first glance. Moving from these illustrations to our conceptual review, we then discuss the diverse manifestations of each tension within both individual-level and collective-level pursuits of integrated care.

### Tension 1. An integrated knowledge base: the need to reconcile lay and professional knowledge

**Illustration 1. Tapping into users’ experiences: ‘person-centered meals’**

In response to complaints about the meals served in the nursing home, the responsible manager joined a client-council meeting—after all, the clients themselves knew best what was needed for improvement. As he tried to capture clients’ views and insights, however, the manager’s questions were heavily shaped by his own professional knowledge of how to improve people’s ‘food experience’, e.g.: ‘How do you like the current food presentation and plate lay-out?’ Ridiculing the question, one resident jokingly replied: ‘Maybe they can put a flower on our trays…!’ Expressing his relief in discovering a lack of major complaints, the manager soon left the meeting. It was only after he left that people started sharing their dissatisfaction and how they thought the meals could be improved.

(Based on the first author’s field notes)

What counts as legitimate knowledge within organizational processes and how is such knowledge generated and utilized? By definition, the pursuit of person-centered integrated care involves the amalgamation of multiple sources of knowledge [48]. The emergence of citizens as active participants in care integration can be seen as an attempt to more explicitly include lay knowledge in the organization of services. Within *individual care trajectories*, such efforts may include attempts to more actively engage with care users (or their significant others) and their situation-specific knowledge that ‘rests on their own particular experience’ [47]. Similarly, at more collective *governance levels*, citizens or users may contribute ‘with local perspectives, values and attitudes that are not based on expert or experiential knowledge […] but rather based on civic knowledge and the experience generated from membership and participation in particular communities’ [47]. At either level, active engagement with lay insights often stems from a recognition of the limitations of ‘formal’ or ‘expert’ knowledge. Reflecting a broader trend towards a ‘pluralisation of knowledge’ [49], such engagement may catalyze the movement towards person-centered and population-based services.

Integrating these different sources of knowledge, however, does present specific challenges. As the illustration above exemplifies, attempts to ‘tap into’ user knowledge and experience remain highly constrained when these attempts are organized to ‘fit’ within established organizational systems or professional frames of reference. While such attempts at engagement may end up legitimizing established systems or managerial decisions, they often do not contribute to an actual pluralization of knowledge and perspectives (see also, e.g., Dedding and Slager’s [50] reflections on the limitations of institutionalized client councils). Moreover, professionals may choose to only engage with what they consider to be the ‘correct’ type of lay knowledge, i.e., they may actively select, educate and socialize lay participants and, in the process, ‘professionalize’ those who participate [51]. Against this background, even when citizens are actively engaged, their insights may not necessarily provide an effective alternative to established ‘expert’ knowledge bases.

Consequently, despite the fact that lay insights have the potential to constitute a counterpoise to the privileging of professional and/or ‘formalized’ knowledge [49], the fulfilment of such potential is far from self-evident. To be clear, we do not claim the inherent superiority of any one of these forms of knowledge over the other and we certainly do not argue for a general ‘de-professionalization’ of care [52]. Nonetheless, we do argue for increased reflexivity among policy makers, managers and practitioners when balancing and integrating different, sometimes competing sources of knowledge. Without such reflexivity, these actors’ established frames of reference are likely to prevail, even when they deliberately try to engage with lay knowledge and perspectives.

### Tension 2. Organizing for participation: the need to reconcile local and central coordination

**Illustration 2. The necessity of (not) setting boundaries: challenging an ‘open’ process**

Facing empty rooms and a compromised financial situation, the provider organization’s leadership made a clear statement: continuing the rural care home’s operations was only possible with the active participation of local citizens. What such participation meant for local service governance, however, was less clear and remained subject to ongoing debate. During the initial meetings between citizens and employees, the organization’s regional manager emphasized the ‘openness’ of this joint trajectory. He explicitly refused to ‘decide ahead of time what things will look like [in the future]’. In the same vein, his colleague scolded the usual tendency to ‘formulate SMART objectives’ instead proposing to ‘embark on a journey together’ with citizens, without knowing where they would end up. Their colleague from the logistics department, however, grew nervous and wished his superiors would set clear targets: ‘At what point do we say, “We can’t continue like this, we need to close the facilities?” […] We need to be more specific.’

(Based on the first author’s field work – also see [11])

How to effectively organize participatory processes of care integration? Generally speaking, successful care integration is associated with horizontal mechanisms of coordination [19]—both within individual organizations and within the broader care systems that encompass them. Hierarchical control tends to be seen as a barrier for alignment across organizational and professional boundaries [1,53,54]. Similarly, scholars have demonstrated that participatory processes tend to be more effective within decentralized and less standardized organizational environments [38,55]. In fact, citizen participation by definition entails a degree of decentralization. Within *individual care trajectories*, for example, user-participation initiatives around often attempt to move away from hierarchical provider-patient relationships. Such initiatives implore care professionals to support their users to ‘live well’ while also taking into consideration their medical, social and psychological conditions as well as other aspects of their personal situation (work, housing, social network, etc.). Because doing so requires continuous adjustment, both to an individual’s particular (and potentially unstable) situation and to a vast diversity of individual care-user opinions regarding the meaning of ‘living well’ [36], these professionals must practice flexibility. Similarly, on more-collective levels of *service governance*, organizational hierarchies may prompt managers to constrain the space for citizen participation when facing systemic requirements and responsibilities [38]. On both levels, the efforts of participating citizens are arguably more ‘difficult to steer and control’ [12] than the efforts of ‘formal’ institutionalized actors who are subject to hierarchical governance. As a result, citizen participation and flexible horizontal coordination seem to be two sides of the same coin [56].

At the same time, the literature on integrated care and citizen participation also make a contradicting observation. Scholars have pointed to the limitations of decentralization and the inescapability of some degree of hierarchy [1]. Paradoxically, while often being portrayed as a pathway towards integration, decentralization is also associated with the negative consequences of fragmentation, as it may cultivate division within complex organizations and, subsequently, ‘interfere with efficiency and quality goals’ [57]. Solely relying on horizontal alignment among local actors without applying hierarchical control or market-based incentives may lead to a lot of ‘talk’ in local committees but no ‘action’ [58]. Moreover, even without imposing centrally-formulated standards and protocols to steer the behavior of care professionals, local actors must still account for the quality of their work on more collective levels, particularly if central actors (often governments) retain overall responsibilities over the care system. Such accountability requirements often act as implicit disciplinary frameworks, which unavoidably steer local practices and, as a result, effectively recentralize decentralized ways of working [33]. Paradoxically, it seems almost inevitable (and often desirable) that decentralization—which provides the flexibility required for successful care integration and citizen participation—should be accompanied with at least some degree of centralization.

Policy makers, managers, professionals and citizens all face the challenge of dealing with a push for both local alignment and central coordination, sometimes leaving space for ‘bottom-up’ flexibility and mutual adjustment while at other times enforcing ‘top-down’ alignment. In pursuits of integrated care, we consequently contend, the question of ‘who is in control of what’ should be made explicit and needs to be actively negotiated across all levels of integration. Continuing to see decentralization as a panacea serves to obscure, rather than answer, this question.

### Tension 3. Finding your partner: citizens’ diversity and their formation as participants

**Illustration 3. The fragmentation of participatory integration: grasping ‘the community’**

The organization’s management had decided to host a meeting with citizens at a local church’s community hall, a venue right in the middle of town. This, they hoped, would stimulate local citizens’ feeling of ownership in the process, symbolically conveying that the challenges faced by the care home were of key concern to both the organization and the local community. A few days before the meeting was to commence, however, management discovered that a considerable number of the town’s inhabitants belonged to a different church and may refuse to attend a meeting at this location. Facing such fragmentation within the local community, they eventually decided to organize the meeting at their own venue after all.

(Based on the first author’s field work)

Who exactly *are* the ‘citizens’ who are supposed to emerge as partners in care integration? While citizen participation has become an increasingly prominent imperative for service organization, references to ‘citizens’, ‘the community’ or ‘the public’ often remain vague [7,59,60]. Participating citizens are generally neither ‘institutionally, vocationally nor financially bound’ [12]. In fact, dealing with such ambiguity seems to be a defining aspect of citizen involvement in care integration. When pursuing community participation in *service governance*, for example, the lack of a clear and institutionalized ‘local infrastructure’ [11] in such communities fuels such ambiguity: to what extent are those who actually participate representative of a certain population or a particular viewpoint? A willingness to participate in the first place may also differ among citizens, especially if participation means that they are granted new responsibilities over the provision of local services. In order to grasp such diverse perspectives, the usual policy ideal is to involve either a wide range of people or ‘all relevant stakeholders’ [61]. Within *individual care trajectories*, actors face a similar diversity. For example, while ‘the importance of involving informal caregivers is emphasized in official documentation’ [62], in practice it is considerably less clear-cut who these informal caregivers are and with what they can or may want to be engaged. The category of ‘informal caregivers’ is not only extremely diverse, but informal caregiving networks may also be unstable and evolve in unexpected ways [44,63]. As such, and in sum, across these different levels, citizen engagement comes with the challenge of dealing with the diversity and diffuseness of citizens as potential partners—each of which has his or her own perspective on whether (and, if so, how) they can or want to contribute to the provision of local care services.

At the same time, concerns about citizen diversity and participants’ representativeness are easily ‘displaced by more practical considerations about the challenge of getting a(ny) group of individuals who might reasonably be considered to constitute a public engaged in the process’ [64]. Citizen participation needs to be operationalized in order to ‘get it done’ in practice. In the context of efforts aimed at participatory service governance, this means that citizens must be ‘constituted as actors’ [65] in order to actually participate. In this process, it may be tempting and pragmatic to only work with those ‘archetypal active citizens’ who are already constructive and willing to cooperate [60]; however, this effectively excludes the participation of more-critical citizens in the process [65]. Accommodating diversity can also be challenging within individual care trajectories. For example, even in a setting in which professional nursing home staff actively voiced their support of family involvement, ‘staff members were strongly focused on work routines, and families were expected to fit in’ [6]. Faced with citizens’ diverse and diffuse character, officials and professionals inevitably try to structure citizen involvement, i.e., they aim to ‘fix’ their diverse and diffuse natures in their attempts to make participation possible and ‘manageable’.

The need to enable citizens to ‘act’ as concrete actors—which inevitably results in the inclusion of some and the exclusion of others in the process—constitutes a challenging-but-inescapable aspect of participatory care integration. It requires a balancing act: the need to, on the one hand, structure citizen contributions to effectively feed into established organizational processes and responsibilities (e.g., clinical decision-making or service design) while, on the other hand, sufficiently accommodating citizens’ inherent diversity and diffuseness.

### Tension 4. Integrating interests: the need to reconcile citizens’ and organizations’ concerns

**Illustration 4. When concerns collide: partnership vs. financial pressure**

Earlier in the care home’s trajectory, the organization’s previous director had decided that the care home would stay open in spite of current losses. As a major advocate of participatory service design, he concluded that honouring citizens’ interests to keep the facility open outweighed the importance of the organization’s compromised financial status. After years of positive returns, he declared it ‘justifiable to invest when times are harder’. Now that a new director had taken office, however, the organization’s core mission was redefined in favour of a more medicalized approach to care provision, which effectively marginalized citizens’ influence in the process. As a result, the new director considered the same competing issues and arrived at an opposite conclusion: the care home would need to close. Participating citizens, whose initial engagement was supposed to prevent ‘unilateral decision-making’, were only informed about the decision several months after it had been made.

(Based on the first author’s field work – also see [11])

How can an organization effectively align its own interests with those of the people they are supposed to serve? As a counterpoise to the commercialized, bureaucratized and professionalized nature of care services, participation is often pursued in an attempt to attune care systems to the concerns of potential users and their broader communities. On the level of *service governance*, citizen participation is seen as a strategy designed to integrate ‘community values into local decision-making processes’ [66]. Similarly, within *individual care trajectories*, user participation can help to align services with ‘what matters to people’ and with their abilities ‘to shape their own lives’ [36]. Across the various levels of integration—both around individual users and on more-collective levels—participation may, therefore, improve the ‘fit’ between care services and the concerns of users, informal caregivers and other citizens. Nevertheless, the crux of the question remains: What needs to happen when citizens’ concerns are at odds with the objectives and interests that prevail within the organizations that provide, commission or regulate care [67]?

Because citizen participation is always just one policy imperative among many others, engaged citizens may still end up playing a marginal role within decision-making processes [11,68,69]. Professionals, managers and policy makers continuously need to weigh the relative importance of competing interests. Within a market-based system, for example, the need to build long-term relationships with participating citizens may conflict with the need to remain competitive and to ‘capture’ customers [70,71]. Similarly, within individual care trajectories, both user preferences and informal caregiver involvement may at times be at odds with professional norms and guidelines, for example, in terms of personal care, food safety or medication delivery [72]. Faced with such complex webs of competing principles, different employees of a single organization may strike different balances when weighing citizen concerns against logistical, financial, medical-professional or other considerations. Harbouring different views on their organization’s key mission, employees of a care facility may disagree on whether they are mostly a medical service provider (which reinforces a more-hierarchical provider-client relationship) or whether they primarily seek to support citizens’ broader wellbeing (which reinforces a more egalitarian partnership) [11]. Aligning such disparate normative frameworks is often a contentious endeavour.

For our part, we see this potential for conflict and controversy as not only intrinsic to participation, but also as not necessarily a bad thing. If actors’ competing perspectives on ‘good’ or ‘appropriate’ care (and how this should be managed) are neither actively juxtaposed nor, subsequently, aligned within an organisation however, then there is a risk of participation getting ‘compartmentalized’ [7] and boxed off; i.e., of it being seen as a ‘project’ or even a ‘hobby’ of a designated department or staff member. In such cases, the potential conflict between disparate concerns persists but is left unaddressed when, in fact, such conflict needs to be dealt with, not ignored, for participation to be effective.

## Discussion

In this paper, we have zoomed in on the organizational tensions that we consider intrinsic to participatory efforts at care integration but that often remain unaddressed within integrated care literature. By focusing on these tensions, we are able to contribute to our understanding of why such efforts may fall short of their ambition to develop more person-centered and population-based services. In particular, we have highlighted the essential and ongoing challenge inherent in the need to align the technical-rational systems through which care services are organized with users’ diverse and diffuse life-worlds; the pursuit of which may affect established distributions of control and responsibilities. By placing this notion front and center, we generate a more realistic outlook on the opportunities, limitations and pitfalls of citizen participation in care integration. Below, we will now discuss two key implications for the field of integrated care and propose avenues for future research.

First and foremost, our discussion demonstrates that the imperative of putting ‘the individual at the centre of all interventions’ [1] often turns out to be a somewhat misleading metaphor within integrated care practices. The position of users and other citizens in integrated care is inherently more ambiguous, even when their active participation is considered an explicit objective. Suggesting that they are ‘in the driver’s seat’ assumes both that their perspectives and concerns can be dealt with in isolation and that they are equipped with the capacity and opportunity to steer or govern. In turn, these assumptions neglect to recognize the trade-offs that are inherent to participatory service governance (i.e., the need to reconcile: (1) lay and professional knowledge, (2) local alignment and central coordination, (3) citizens’ diversity and their formation as participants, and (4) the concerns of both citizens and organizational actors). By disregarding these tensions, citizens may, paradoxically, wind up in a ‘central’-but-still-marginal position, i.e., that of being passive recipients that care is integrated *for*.

Nevertheless, participation can make a key contribution to care integration—but it requires an explicit focus on the organizational tensions to which it inherently gives rise. Therefore, we propose the following:

*Proposition 1: Because dealing with the tension between* (*a*) *the various technical-rational systems through which care services are organized and* (*b*) *people’s diffuse life-worlds is both an ongoing challenge and a key objective of care integration, such integration requires a collaborative effort; not just among professionals and other institutional stakeholders, but also including users and other citizens as active participants. When citizens are allegedly placed ‘in the driver’s seat’ or, alternatively, treated as passive recipients of managerial or professional attempts at integration, this tension is easily overlooked or left unaddressed*.

As a second contribution, our discussion highlights the importance of approaching integration as a dynamic, ongoing process and not ‘merely’ as an issue of service design. Rather, the deep-seated tensions presented by participation and care integration require the continuous navigation of multiple legitimate-but-contradictory objectives (e.g., the need to create space for local alignment while still meeting centrally-formulated operational and governance standards). Within extant literature, this processual character of integration has not always been sufficiently recognized. Integrated care is often conceptualized as something that is pursued ‘by design’ [73]. Take, for example, the often-cited definition of integrated care as ‘a coherent set of methods and models […] designed to create connectivity, alignment and collaboration’ [19]. By concealing the intricate challenge of juggling disparate considerations within dynamically evolving contexts—both between and within the organizations and the communities involved—a focus on integration ‘by design’ overemphasizes integration as a linear process. Bearing in mind the ‘unbound’ and ‘fundamentally uncertain’ character of citizen participation [12], these concerns become even more pressing. Moreover, the literature often treats integration as a care system’s ideal end state, i.e., one in which users ‘experience services as “seamless”’ [1]. In pursuing a ‘seamless’ experience and process of care delivery, however, professionals and managers may try to keep friction ‘backstage’, which (often) unintentionally restricts users’ and other citizens’ potential contributions to the relevant issues at hand. Instead, we argue for a conceptualization that highlights integration’s processual character. We therefore propose:

*Proposition 2: Because the pursuit of participatory care integration reinforces the need to approach integration as an ongoing activity and process, not as a care system’s ‘*(*end*) *state’, the need to align systems with users’ dynamically evolving life-worlds requires continuous attention to the friction that inherently emerges in that process. If integration is approached as a ‘one-off’ or static effort at system redesign, participation is bound to wither*.

In sum, care integration is not only dependent on system integration. It is also dependent on ongoing efforts to align (a) the professional, organizational and institutional frameworks through which practitioners, managers and policy makers structure their work with (b) the dynamics and diffuseness of users’ and citizens’ life-worlds. From this perspective, the continued participation of not only (potential) care users, but also of their significant others and other citizens, is a requirement for attuning care services to what matters most in people’s lives—to supporting their health and wellbeing. As such, we call on scholars, policy makers and practitioners of integrated care to actively deal with, instead of ignore, the tensions that inherently emerge when seeking to align these systems and life-worlds.

Adopting such a conceptualization of care integration opens up relevant avenues for future research. In particular, we would like to highlight the possibilities for examining an under-investigated aspect of integrated care: power dynamics. In one of his five seminal ‘laws of integration’, Leutz states that ‘[t]he one who integrates calls the tune’ [74]. Indeed, efforts to engage citizens as active ‘integrators’ are often presented as attempts to give citizens more control over the services they may potentially use. Nonetheless, the field of integrated care is relatively limited in paying explicit attention to the power dynamics triggered by such pursuits—recent work by Kaehne [75] being a notable exception. Scholarship outside the field, however, certainly proves the relevance of a more power-sensitive analysis of care integration (e.g., see [76,77]). This becomes even more pressing when it comes to citizen participation. Participatory efforts at care integration constitute a balancing act—one in which the balance is easily skewed towards individual organizations’ concerns at the expense of citizens’ interests [11]. Ample case studies illustrate how citizen participation can be made instrumental to the interests of both professionals and public service agencies and how it can be marginalized within decision-making processes [11,36,78,79]. Although professionals, managers and policy makers may consciously pursue citizen empowerment, the pressures of institutional, organizational or professional obligations may inadvertently end up disempowering citizens in the process [59]. As such, we consider the critical scrutiny of the power dynamics of citizen participation in care integration an ethical imperative. When doing so, the four tensions described in this paper (i.e., those that result from the need to reconcile (1) lay and professional knowledge, (2) local alignment and central coordination, (3) citizens’ diversity and their formation as participants, and (4) the concerns of citizens and organizational actors) may serve as a relevant research guide by pointing out the areas in which such power dynamics are likely to be found.

## Concluding remarks

Inspired by our own research and based on a critical review of key publications in the field, we have sought to further the debate on citizen participation within care integration. If we approach care not only as a product but also as something that is constituted within the interactions between care users, lay caregivers and professionals [22,71], then co-production inevitably lies at the heart of all care delivery [80]. Illustrated by the field’s predominantly professional and organizational characterization of integration [2,19], we believe that this notion has yet to be fully reflected within established approaches to integrated care. In our view, in order to realize ambitions of person-centeredness and population-based care, we should stop treating citizen participation as an add-on to an otherwise professional, organizational and institution paradigm. Likewise, we must refrain from idealizing participation as an easy remedy or an undisputed objective. In practice, the intricate tensions between systems and life-worlds are an inevitable part of the process of pursuing participatory care integration.
